# Antimicrobial Drug–Resistant *Escherichia coli* from Humans and Poultry Products, Minnesota and Wisconsin, 2002–2004

**DOI:** 10.3201/eid1306.061576

**Published:** 2007-06

**Authors:** James R. Johnson, Mark R. Sannes, Cynthia Croy, Brian Johnston, Connie Clabots, Michael A. Kuskowski, Jeff Bender, Kirk E. Smith, Patricia L. Winokur, Edward A. Belongia

**Affiliations:** * Minneapolis Veterans Affairs Medical Center, Minneapolis, Minnesota, USA; † University of Minnesota, Minneapolis, Minnesota, USA; ‡ University of Minnesota, Saint Paul, Minnesota, USA; § Minnesota Department of Health, Saint Paul, Minnesota, USA; ¶ University of Iowa, Iowa City, Iowa, USA; # Iowa City Veterans Affairs Medical Center, Iowa City, Iowa, USA; ** Marshfield Clinic Research Foundation, Marshfield, Wisconsin, USA; 1 Current affiliation: Park Nicollet Clinic, Saint Louis Park, Minnesota, USA

**Keywords:** Escherichia coli infections, antimicrobial drug resistance, virulence, phylogenetics, PCR, molecular fingerprinting, foodborne disease, poultry, zoonoses, research

## Abstract

The food supply, including poultry products, may transmit antimicrobial drug–resistant *Escherichia coli* to humans. To assess this hypothesis, 931 geographically and temporally matched *E. coli* isolates from human volunteers (hospital inpatients and healthy vegetarians) and commercial poultry products (conventionally raised or raised without antimicrobial drugs) were tested by PCR for phylogenetic group (A, B1, B2, D) and 60 virulence genes associated with extraintestinal pathogenic *E. coli*. Isolates resistant to trimethoprim-sulfamethoxazole, quinolones, and extended-spectrum cephalosporins (n = 331) were compared with drug-susceptible isolates (n = 600) stratified by source. Phylogenetic and virulence markers of drug-susceptible human isolates differed considerably from those of human and poultry isolates. In contrast, drug-resistant human isolates were similar to poultry isolates, and drug-susceptible and drug-resistant poultry isolates were largely indistinguishable. Many drug-resistant human fecal *E. coli* isolates may originate from poultry, whereas drug-resistant poultry-source *E. coli* isolates likely originate from susceptible poultry-source precursors.

## Abstract 2

Similarities were found between drug-resistant *E*. *coli* from humans and poultry products.

Acquired resistance to first-line antimicrobial agents increasingly complicates the management of extraintestinal infections due to *Escherichia coli*, which are a major source of illness, death, and increased healthcare costs (*1*–*4*). One suspected source of drug-resistant *E*. *coli* in humans is use of antimicrobial drugs in agriculture. This use presumably selects for drug-resistant *E. coli*, which may be transmitted to humans through the food supply (*5*–*7*). Supporting this hypothesis is the high prevalence of antimicrobial drug–resistant *E*. *coli* in retail meat products, especially poultry (*8*–*11*), and the similar molecular characteristics of fluoroquinolone-resistant *E*. *coli* from chicken carcasses and from colonized and infected persons in Barcelona, Spain, in contrast to the marked differences between drug-susceptible and drug-resistant source isolates from humans (*12*).

To further assess the poultry-human connection, we used molecular typing to characterize drug­-resistant and drug-susceptible *E*. *coli* isolates from feces of human volunteers or newly hospitalized patients in Minnesota and Wisconsin and from poultry products sold or processed in the same region. Resistance phenotypes of interest include trimethoprim-sulfamethoxazole (TMP-SMZ), quinolones/fluoroquinolones, and extended-spectrum cephalosporins. These agents are used for treatment of human *E*. *coli* infections. These drugs (or congeners) are also used in poultry production (e.g., each year in the United States an estimated 1.6 billion broiler eggs or chicks receive ceftiofur [*13*]); *E*. *coli* isolates resistant to these drugs are found in poultry. We examined according to phylogenetic group distribution and virulence gene profile, whether drug-resistant human isolates more closely resemble susceptible human isolates, which is consistent with acquisition of resistance within humans, or instead resemble poultry isolates, which is consistent with foodborne transmission of poultry-source organisms to humans. We also examined whether poultry-source resistant and susceptible isolates are similar, which is consistent with emergence of resistance on farms under selection from agricultural use of antimicrobial drugs.

## Methods

### Participants and Bacterial Strains

Human fecal samples were collected from 622 adults newly admitted to local hospitals in 4 rural communities in Minnesota (Willmar) or Wisconsin (Eau Claire, La Crosse, and Marshfield) and from 100 healthy self-identified vegetarians in these and nearby communities (*14*). Hospital patients were recruited from June 2002 through May 2003, vegetarians during the first 6 months of 2004. Fecal samples were collected by study personnel by using rectal swabs (hospital patients) or by the participants (vegetarians). To prevent isolation of hospital-acquired flora, inpatients samples were collected within 36 hours of hospital admission. Guidelines of the authors’ institutions regarding use of human subjects were followed in this study. The relevant institutional review boards reviewed and approved the protocol. All participants provided informed consent.

A total of 180 retail poultry products (155 chicken and 25 turkey) were sampled (*14*). Conventional brands were purchased systematically from all food markets in the 4 primary study communities from May 2002 through May 2003, with 40 retail items obtained per community (total 160 items). These represented at least 18 plants in 11 states. Twenty samples with labels indicating that the poultry were raised naturally or without antibiotics were purchased in or near the study communities in August 2004. Additionally, 40 freshly slaughtered chicken carcasses from local farmers who raised chickens naturally or without antibiotics were obtained during plant inspections by the Minnesota Department of Agriculture from September 2003 through August 2004. The latter 2 groups of chickens, designated “no antibiotics,” were confirmed to have been raised without antibiotics, based on the product label or by contacting the manufacturer or distributor.

### Sample Processing

Human fecal samples were suspended and poultry samples and carcasses were massaged in nutrient broth, which was then incubated overnight at 37°C and stored as aliquots at –80°C in glycerol (*14*). Portions of these frozen stocks were transferred to vancomycin-supplemented (20 mg/L) Luria-Bertani broth. After overnight incubation, these broths were plated directly onto modified Mueller-Hinton (MMH) agar (Amyes medium) (*10*) with and without ciprofloxacin (4 mg/L) and (separately) nalidixic acid (32 mg/L), and were then incubated overnight. Samples of these Luria-Bertani broths containing vancomycin were placed in MMH broths supplemented individually with TMP-SMZ (4 mg/L TMP plus 76 mg/mL SMZ), cefoxitin (10 mg/L and 32 mg/L), and ceftazidime (10 mg/L and 32 mg/L). After overnight incubation, these broths were plated onto MMH agar plates supplemented with the corresponding agent (same concentrations) for overnight incubation. Colonies resembling *E*. *coli* were identified by using the API-20E System (bioMérieux, Marcy-l’Etoile, France).

### Susceptibility Testing

At least 1 *E*. *coli* colony was randomly selected from each MMH agar plate and tested for disk susceptibility to 24 antimicrobial agents by using Clinical Laboratory Standards Institute (CLSI)–recommended methods, interpretive criteria, and reference strains (*15*). For isolates resistant to TMP-SMZ, nalidixic acid, or ciprofloxacin, the MIC was determined by Etest (AB-Biodisk, Sona, Sweden) according to the manufacturer's directions. Isolates from cefoxitin- and ceftazidime-supplemented plates underwent broth dilution MIC determinations with cefotaxime and ceftazidime regardless of disk test results. Isolates were classified as resistant to TMP-SMZ if the TMP MIC was >4 mg/L and the SMZ MIC was >76 mg/L, to quinolones if the nalidixic acid MIC was >32 mg/L, to fluoroquinolones if the ciprofloxacin MIC was >4 mg/L, and to extended-spectrum cephalosporins if the MIC to either cefotaxime or ceftazidime was >16 mg/L. The latter threshold corresponds with intermediate susceptibility per CLSI criteria and includes isolates with potentially clinically relevant reduced susceptibility. Because of the small number of isolates within each resistance phenotype, isolates were classified as resistant if they met any of these resistance criteria. Isolates that did not meet any of these resistance criteria were classified as susceptible, even though they may have had reduced susceptibility to other drug classes.

From each sample, 1 colony of each resistance phenotype (TMP-SMZ, quinolones, fluoroquinolones, extended-spectrum cephalosporins) and 1 susceptible isolate, as available, were selected. If multiple isolates from a given sample exhibited similar disk diffusion susceptibility profiles, genomic profiles as generated by using random amplified polymorphic DNA (RAPD) analysis were compared in the same gel (*12*). One representative of each unique RAPD genotype (as determined by visual inspection) was arbitrarily selected for further analysis.

### Phylogenetic Analysis and Virulence Genotyping

All isolates were categorized as to major *E. coli* phylogenetic group (A, B1, B2, or D) by a multiplex PCR-based assay (*16*) (Table 1). Genes encoding proven or putative virulence factors of extraintestinal pathogenic *E*. *coli* (ExPEC) were detected in a sequential fashion. All isolates were screened for 5 ExPEC-defining virulence genes and *hlyD* (hemolysin). Isolates were operationally defined as ExPEC if ≥2 of the following were present: *papA* and/or *papC* (P fimbriae structural subunit and assembly), *sfa/focDE* (S and F1C fimbriae), *afa/draBC* (Dr binding adhesins), *iutA* (aerobactin system), and *kpsM* II (group 2 capsule) (*8*). All ExPEC isolates were then tested for 60 ExPEC-associated virulence genes and alleles thereof. Testing was conducted by using 2 independently prepared lysates of each isolate and established PCR-based methods (*12*,*17*). Isolates from various source groups (e.g., hospital volunteers, conventionally raised poultry) were tested in parallel to avoid cohort effects. The virulence score was the number of virulence genes detected adjusted for multiple detection of the *pap*, *sfa/foc*, and *kps* operons (*12*).

**Table 1:** Bacterial traits by source and antimicrobial drug resistance in 931 *Escherichia coli* isolates from human feces and poultry products, Minnesota and Wisconsin, 2002–2004*

Trait†	Prevalence, no. (%)				p value‡		
	Total (n = 931)	Human, susceptible (n = 460)	Human, resistant (n = 70)	Poultry (n = 401)	HS vs. HR	HS vs. all poultry	HR vs. all poultry
Group A	252 (27)	96 (21)	23 (33)	133 (33)		≤0.001	
Group B1	186 (20)	79 (17)	11 (16)	96 (24)			
Group B2	234 (25)	178 (39)	13 (19)	43 (11)	≤0.001	≤0.001	
Group D	259 (28)	107 (23)	23 (33)	129 (32)		≤0.01	
*papA*	124 (13)	98 (21)	6 (9)	20 (5)		≤0.001	
*papC*	163 (18)	100 (22)	10 (14)	53 (13)		≤0.001	
*sfa/focDE*	69 (7)	65 (14)	2 (3)	2 (0.5)	≤0.01	≤0.001	
*afa/draBC*	19 (2)	14 (3)	5 (7)	0 (0)		≤0.001	≤0.001
*iutA*	361 (39)	93 (20)	32 (46)	236 (59)	≤0.001§	≤0.001§	
*kpsM* II	288 (31)	195 (42)	23 (33)	70 (17)		≤0.001	≤0.01
*hlyD*	71 (8)	64 (14)	2 (3)	4 (1)	≤0.01	≤0.001	
ExPEC	249 (27)	147 (32)	20 (29)	82 (20)		≤0.001	

*Data are for the total population. Susceptible, susceptible to trimethoprim-sulfamethoxazole, nalidixic acid (quinolones), and ceftriaxone or ceftazidime (extended-spectrum cephalosporins), regardless of other possible drug resistance; resistant, resistant to 1 of the following: trimethoprim-sulfamethoxazole, nalidixic acid (quinolones), and ceftriaxone or ceftazidime (extended-spectrum cephalosporins). †Groups A, B1, B2, and D, major *E. coli* phylogenetic groups; *papA* and *papC*, P fimbriae structural subunit and assembly; *sfa/focDE*, S and F1C fimbriae; *afa/draBC*, Dr binding adhesins; *iutA*, aerobactin system; *kpsM* II, group 2 capsule; *hlyD*, α-hemolysin; ExPEC, extraintestinal pathogenic *E. coli* defined by presence of ≥2 of *papA* and/or *papC* (counted as 1), *sfa/focDE*, *afa/draBC*, *iutA*, and *kpsM* II. ‡By Fisher exact test. Values are shown only where p≤0.01. HS, susceptible isolates from humans; HR, resistant isolates from humans. Because drug-resistant and drug-susceptible poultry isolates showed only 1 significant difference (for *iutA*), they were combined into an all-poultry group. §Negative association.

### Statistical Methods

The unit of analysis was the individual isolate. Comparisons of proportions were tested by using Fisher exact test (2-tailed). Comparisons of virulence scores were tested by using Mann-Whitney U test (2-tailed exact probability). Principal coordinates analysis (PCA), also known as metric multidimensional scaling, is a multivariate statistical technique used to provide a simpler, low-dimensional graphic summary of the similarity between multiple samples (e.g., isolates) across multiple loci (*18*). New axes for plotting the isolates are derived from a data matrix of estimated dissimilarities between isolates. The first 2 principal coordinates, which account for the most variance, are used to plot the data. The distances between points in the plot represent isolate similarity. The dimensions represented by the (statistically uncorrelated) axes have no intrinsic meaning, i.e., they have no units. Using GenAlEx6 (*19*), we applied PCA to the screening dataset (all isolates) and the extended virulence profile dataset (ExPEC isolates) as a way to collapse the multiple variables for simplified among-group comparisons. For each PCA, results for each isolate from the first 2 PCA axes were used in multiple analysis of variance (MANOVA) to test for among-group differences. These values also were plotted to spatially represent the degree of separation or overlap of isolates on the 2-axis plane. For the ExPEC isolates, pairwise similarity relationships according to extended virulence profiles and phylogenetic group were used to construct a dendrogram according to the unweighted pair group method with arithmetic averages (*20*). The criterion for statistical significance throughout was p≤0.01 to account for multiple comparisons.

## Results

### Isolation of Drug-Resistant and Drug-Susceptible *E*. *coli*

Selective processing of 942 human fecal and poultry samples yielded 931 unique *E*. *coli* isolates, which constituted the study population. Of the 931 isolates, 530 (57%) were from human volunteers and 401 (43%) from poultry products. Of the human isolates, 456 (86%) were from hospital patients and 74 (14%) from vegetarians. Of the poultry isolates, 289 (72%) were from conventionally raised retail poultry and 112 (28%) from poultry raised without antibiotics. The median number of unique *E*. *coli* isolates per sample was 1 for human fecal samples and 2 for poultry (range 1–4 for both).

Overall, 331 isolates (70 human, 261 poultry) were classified as resistant on the basis of reduced susceptibility to TMP-SMZ, quinolones/fluoroquinolones, and extended-spectrum cephalosporins. The remaining 600 isolates (460 human, 140 poultry) were susceptible to all these drug classes and were classified as susceptible (regardless of other possible drug resistance). The resistant isolates were distributed by resistance phenotype as follows: TMP-SMZ, 154 (47 human, 107 poultry); quinolones, 115 (26 human, 89 poultry); and extended-spectrum cephalosporins, 114 (14 human, 100 poultry). The 7 fluoroquinolone-resistant isolates (5 human, 2 poultry) were analyzed within the quinolone-resistant group.

### Phylogenetic Distribution and Prevalence of ExPEC-defining Markers

The initial screening showed the 931 isolates to be fairly evenly distributed among the 4 major *E*. *coli* phylogenetic groups (20%–28% per group). However, they had various prevalences (2%–39% each) of the screening ExPEC virulence genes (Table 1). Overall, 27% of the isolates qualified as ExPEC by having ≥2 of the 5 ExPEC-defining markers (Table 1).

For enhanced resolution of similarities and differences, the 243 available ExPEC isolates underwent extended virulence genotyping for 60 ExPEC-associated virulence genes. All but 6 of these traits were detected in ≥1 isolate each, with prevalences ranging from 0.4% to 98% (Table 2).

**Table 2:** Bacterial traits by source and antimicrobial drug resistance in 243 extraintestinal pathogenic *Escherichia coli* (ExPEC) isolates from human feces and poultry products, Minnesota and Wisconsin, 2002–2004*

Trait†‡§	Prevalence, no. (%)				p value¶		
	Total (n = 243)	Human, susceptible (n = 144)	Human, resistant (n = 20)	Poultry (n = 79)	HS vs. HR	HS vs. all poultry	HR vs. all poultry
Group A	20 (8)	5 (3)	5 (25)	10 (13)	≤0.01#		
Group B1	7 (3)	0	0	7 (9)		≤0.001#	≤0.001#
Group B2	154 (63)	125 (87)	6 (30)	23 (29)		≤0.001	
Group D	62 (26)	14 (10)	9 (45)	39 (49)		≤0.001#	
*papA*	117 (48)	97 (67)	7 (35)	13 (16)	≤0.01	≤0.001	
F10 allele	38 (16)	32 (10)	5 (25)	1 (1)		≤0.001	≤0.001
F16 allele	12 (5)	5 (3)	5 (25)	2 (3)	≤0.01#		≤0.01
F48 allele	21 (9)	21 (15)	0	0		≤0.001	
*papG* III	44 (18)	44 (31)	0	0	≤0.01	≤0.001	
*sfa/focDE*	62 (26)	61 (42)	1 (5)	0	≤0.001	≤0.001	
*sfaS*	35 (14)	33 (23)	1 (5)	1 (1)		≤0.001	
*focG*	13 (5)	12 (8)	1 (5)	0		≤0.01	
*afa/draBC*	15 (6)	11 (8)	4 (20)	0		≤0.01	≤0.001
*iha*	52 (22)	38 (26)	16 (80)	0	≤0.001#	≤0.001	≤0.001
*hra*	108 (44)	67 (47)	2 (10)	39 (49)	≤0.001		≤0.01#
*cnf1*	54 (22)	51 (35)	2 (10)	1 (1)		≤0.001	
*hlyD*	67 (28)	67 (28)	2 (10)	2 (3)	≤0.01	≤0.001	
*hlyF*	73 (30)	28 (19)	1 (5)	44 (57)		≤0.001#	≤0.001#
*sat*	61 (25)	46 (32)	15 (75)	0 (0)	≤0.001#	≤0.001#	≤0.001#
*pic*	34 (14)	30 (21)	0	4 (5)		≤0.01	
*tsh*	131 (54)	113 (78)	3 (15)	15 (19)	≤0.001	≤0.001	
*astA*	48 (20)	7 (5)	1 (5)	40 (51)		≤0.001#	≤0.001#
*iutA*	162 (67)	67 (47)	18 (90)	77 (97)		≤0.001#	
*iroN*	118 (49)	78 (54)	3 (15)	37 (47)	≤0.001		≤0.01#
*fyuA*	199 (82)	138 (96)	17 (85)	44 (56)		≤0.001	
*kpsM* II	215 (89)	137 (95)	16 (80)	62 (78)		≤0.001	
K5 *kpsM*	35 (14)	28 (19)	4 (20)	3 (4)		≤0.001	
*iss*	69 (28)	23 (16)	2 (10)	44 (56)		≤0.001#	≤0.001#
*usp*	144 (59)	127 (88)	6 (30)	11 (14)	≤0.001	≤0.001	
H7 *fliC*	52 (21)	52 (36)	0	0	≤0.001	≤0.001	
*ompT*	184 (76)	131 (91)	9 (50)	40 (51)	≤0.01	≤0.001	
*malX*	152 (63)	134 (93)	7 (35)	1 (14)	≤0.001	≤0.001	

*Susceptible, susceptible to trimethoprim-sulfamethoxazole, nalidixic acid (quinolones), and ceftriaxone or ceftazidime (extended-spectrum cephalosporins), regardless of other possible drug resistance; resistant, resistant to >1 of the following: trimethoprim-sulfamethoxazole, nalidixic acid (quinolones), and ceftriaxone or ceftazidime (extended-spectrum cephalosporins). †Traits are shown that showed p≤0.01 for ≥1 comparison each. Groups A, B1, B2, and D, major *E. coli* phylogenetic groups; *papA*, P fimbriae structural subunit with variants F10, F16, and F48; *papG* III, variant P adhesin; *sfa/focDE*, S and F1C fimbriae; *sfaS*, S fimbriae; *focG*, F1C fimbriae; *afa/draBC*, Dr binding adhesins; *iha*, adhesin-siderophore receptor; *hra*, pathogenicity island marker; *cnf1*, cytotoxic necrotizing factor 1; *hlyD*, α-hemolysin; *hlyF*, variant hemolysin; *sat*, secreted autotransporter toxin; *pic*, autotransporter protease; *tsh*, autotransporter hemagglutinin; *astA*, enteroaggregative *E. coli* toxin; *iutA*, aerobactin system; *iroN*, siderophore receptor; *fyuA*, yersiniabactin receptor; *kpsM* II, group 2 capsule; K5 *kpsM*, *kpsM* II variant; *iss*, increased serum survival; *usp*, uropathogenic-specific protein; H7 *fliC*, flagellar variant; *ompT*, outer membrane protease; *malX*, pathogenicity island marker. ‡Traits that did not show p<0.01 but were detected in ≥1 isolate each include the F7–2, F8, F9, F11, F12, F12, F14, and F15 *papA* alleles, *papC* (P fimbriae assembly), *papEF* (P fimbriae tip pilins), *papG* alleles I and II (both internal and flanking sequences), *afaE8* (variant Dr binding adhesin), *gafD* (G fimbriae), F17 fimbriae, *fimH* (type 1 fimbriae), *clpG* (adhesin), *cdtB* (cytolethal distending toxin B), *ireA* (siderophore receptor), *kpsM* III (group 3 capsule), K1 and K2 *kpsM* II variants, *cvaC* (microcin V), *ibeA* (invasion of brain endothelium), and *rfc* (O4 lipopolysaccharide biosynthesis). §Traits not detected in any isolate include F7–1 and F536 *papA* alleles and K15 *kpsM* II variant. ¶By Fisher exact test. Values are shown only where p≤0.01. HS, susceptible isolates from humans; HR, drug-resistant isolates from humans. Because drug-resistant and drug-susceptible poultry isolates showed no significant differences, they were combined into an all-poultry group. #Negative association.

### Prevalence Comparisons

Phylogenetic group distribution and virulence gene prevalence differed considerably according to source (human versus poultry) and resistance status. This finding is shown in Table 1 for all 931 isolates (screening virulence genes only) and in Table 2 for the 243 ExPEC isolates (extended virulence profiles). Drug-resistant and drug-susceptible human isolates were separately compared with the combined group of all poultry isolates (i.e., all susceptible and resistant). We analyzed poultry isolates as a single group because the distribution of traits was similar in drug-resistant and susceptible poultry isolates; i.e., only 1 trait (*iutA*) was significantly associated with resistance among poultry isolates.

Consistent differences in phylogenetic and virulence gene distribution were evident between groups (Tables 1, 2). First, drug-susceptible human isolates differed considerably from drug-resistant human isolates. Second, drug-susceptible human isolates differed from poultry isolates. Third, although human drug-resistant isolates and poultry isolates exhibited some differences, these were considerably fewer and less extreme than those between drug-susceptible human isolates and poultry isolates. Similar results were obtained in subgroup analyses when isolates from hospital patient fecal samples were compared separately with isolates from conventionally raised poultry or when isolates from fecal samples from vegetarians were compared separately with isolates from poultry raised without antibiotics.

### PCA

PCA was used to concurrently analyze multiple bacterial characteristics. The first PCA was conducted for the total population (n = 931) with the 7 screening virulence genes plus phylogenetic group. According to a 2 × 2 (source × resistance status) MANOVA of the first 2 axes of the PCA (which accounted for 65% of total variance), all 3 independent variables considered (source, resistance status, and interaction term) showed a p value ≤0.001. Accordingly, pairwise comparisons were made between individual source-resistance groups by 1-factor MANOVA. Susceptible human isolates differed (p<0.001) from each of the other 3 groups, whereas the other 3 groups differed marginally from each other. The individual axes supported this conclusion. These axes showed more extreme differences between drug-susceptible human isolates and each of the other 3 groups (p<0.001 for 5 of 6 comparisons) than among the other groups (p>0.01 for 4 of 6 comparisons).

**Figure 1:**
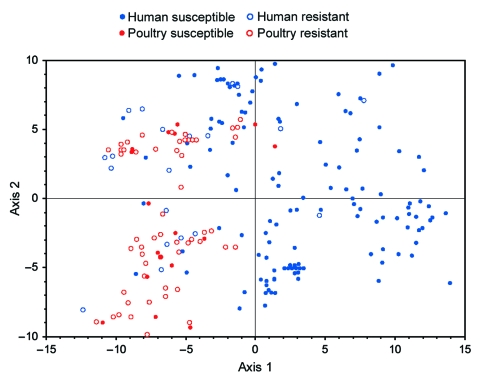
Principal coordinates analysis of distribution of 243 extraintestinal pathogenic *Escherichia coli* isolates from human feces and poultry products, Minnesota and Wisconsin, 2002–2004, on the axis 1–axis 2 plane. Data include extended virulence genotypes (60 traits) and phylogenetic group (A, B1, B2, D). The axes have no units; they reflect the total score for each isolate derived by summing the isolate's partial score for each variable, which is the product of the loading score assigned to the particular variable for a given axis and the isolate's status for that variable. Axis 1 (positive values to right, negative values to left of central vertical line) accounted for 37% of total variance and showed significant differences between susceptible human isolates versus each of the other groups. Axis 2 (positive values above, negative values below central horizontal line) accounted for 20% of total variance and did not show any significant between-group differences. Resistant, resistant to trimethoprim-sulfamethoxazole, nalidixic acid (quinolones), and ceftriaxone or ceftazidime (extended-spectrum cephalosporins). Susceptible, susceptible to all these agents (regardless of other possible drug resistance).

Next, PCA was conducted for the 243 available ExPEC isolates based on all 60 virulence genes plus phylogenetic group. According to an initial 2 × 2 MANOVA of the results from the first 2 PCA axes (which accounted for 57% of total variance), all 3 independent variables (source, resistance status, and interaction term) showed a p value <0.001. Accordingly, pairwise comparisons were made between individual source-resistance groups by 1-factor MANOVA. Susceptible human isolates differed (p<0.001) from each of the other 3 groups, whereas the other 3 groups did not differ significantly from each another. In a plot of the (axis 1–axis 2) plane, drug-susceptible poultry isolates, drug-resistant poultry isolates, and drug-resistant human isolates overlapped and were confined largely to the left half of the grid (negative values on axis 1). In contrast, drug-susceptible human isolates, although overlapping somewhat with these groups, were concentrated principally within the right half of the grid (positive values on axis 1) (Figure 1).

### Aggregate Virulence Scores

**Figure 2:**
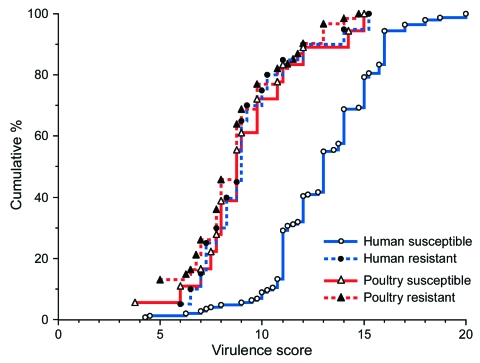
Distribution of virulence factor scores by source and resistance status among 243 extraintestinal pathogenic *Escherichia coli* isolates from human feces and poultry products, Minnesota and Wisconsin, 2002–2004. Resistant, resistant to trimethoprim-sulfamethoxazole, nalidixic acid (quinolones), and ceftriaxone or ceftazidime (extended-spectrum cephalosporins). Susceptible, susceptible to all these agents (regardless of other possible resistances). The virulence scores of the susceptible human isolates are an average of ≈4 points greater than those of the resistant human isolates or poultry isolates.

The various source and resistance groups were also compared for aggregate virulence scores (ExPEC isolates only). According to virulence score distribution, drug-susceptible human isolates (higher scores) segregated widely from the other 3 subgroups (lower scores), which were largely superimposed on each another (Figure 2). Because drug-resistant and drug-susceptible poultry isolates had similar virulence scores, they were combined for statistical analysis. Drug-susceptible human isolates had the highest scores (median 13.0, range 4.25–20.0). Drug-resistant human and poultry isolates had significantly lower scores that did not differ between humans and poultry (median 9.0, range 6.0–15.25, and median 8.75, range 3.75–15.0, respectively; vs. drug-susceptible human isolates, p<0.001). Similar results were obtained when isolates from hospital patient fecal samples were compared separately with the conventionally raised poultry isolates or when isolates from vegetarian fecal samples were compared separately with isolates from poultry raised without antibiotics (data not shown).

### Dendrogram of Extended Virulence Profiles and Phylogenetic Group

**Figure 3:**
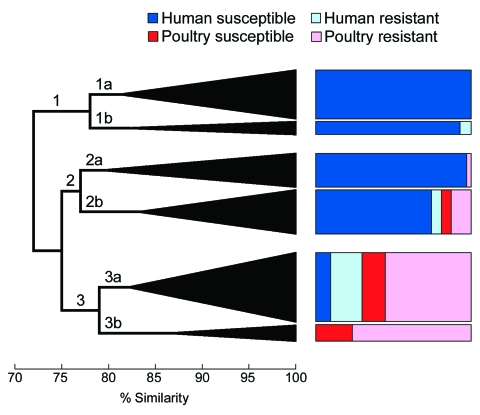
Dendrogram based on extended virulence profiles of 243 extraintestinal pathogenic *Escherichia coli* isolates from human feces and poultry products, Minnesota and Wisconsin, 2002–2004. The dendrogram (shown here in simplified form) was constructed by using the unweighted pair group method with arithmetic averages based on pairwise similarity relationships according to the aggregate presence or absence of 60 individual virulence genes plus phylogenetic group (A, B1, B2, D). Triangles indicate arborizing subclusters. Major clusters 1, 2, and 3, and subclusters 1a, 1b, 2a, 2b, 3a, and 3b are indicated. Colored boxes to right of dendrogram show the distribution (by source group) of constituent members of each subcluster. Resistant, resistant to trimethoprim-sulfamethoxazole, nalidixic acid (quinolones), and ceftriaxone or ceftazidime (extended-spectrum cephalosporins). Susceptible, susceptible to all these agents.

Phylogenetic group and extended virulence profiles among the 243 available ExPEC isolates also were used to construct a similarity dendrogram. The dendrogram showed 3 major clusters, each of which contained 2 prominent subclusters (Figure 3). Isolates were distributed by cluster and subcluster according to source and resistance group; that is, drug-susceptible human isolates accounted for almost all of subclusters 1a, 1b, and 2a. In contrast, drug-resistant human isolates were confined largely to subcluster 3a. Poultry isolates, whether resistant or susceptible, were confined almost entirely to subclusters 2b, 3a, and 3b. Thus, compared with drug-susceptible human isolates, drug-resistant human isolates were significantly more likely to occur within a subcluster, or major cluster, that also contained poultry isolates (p<0.001 for each comparison).

The possible effects of nonindependence among multiple isolates acquired from the same sample were assessed by limiting the analysis to a single isolate per sample, keeping a drug-susceptible isolate (if available) and randomly selecting among multiple drug-resistant isolates where required. This resulted in reduced sample sizes of 681 (total population) and 226 (ExPEC population). The analysis results closely mirrored the pattern of significant findings obtained in the full samples.

## Discussion

In this study, we analyzed the phylogenetic distribution and virulence genotypes of drug-susceptible and drug-resistant *E*. *coli* isolates from human volunteers and poultry products in Minnesota and Wisconsin. We found that drug-resistant human isolates, although overlapping somewhat with drug-susceptible human isolates, were more similar overall to poultry isolates than to drug-susceptible human isolates. In contrast, drug-susceptible human isolates differed from poultry isolates. This relationship was observed consistently with diverse analytical approaches and various stratifications of the population. It suggests that many of the drug-resistant human isolates were more likely to have originated in poultry (or a similar nonhuman reservoir) and to have been acquired by humans when these isolates were already drug resistant, than to have emerged de novo in humans by conversion of drug-susceptible human isolates to drug-resistant isolates.

We also found that, regardless of analytical approach and population analyzed, resistant and susceptible poultry isolates were highly similar. This suggests that the resistant poultry isolates likely derived from antimicrobial drug–susceptible, poultry-source *E*. *coli* by conversion to resistance. This most plausibly would occur within the avian fecal flora under selection pressure from on-farm use of antimicrobial drugs.

Our findings closely resemble those of a recent study of ciprofloxacin-resistant *E*. *coli* from humans and chickens in the late 1990s in Barcelona, Spain (*12*). These data indicate that these relationships remain valid and are applicable in the United States, to additional resistance phenotypes (specifically quinolones, TMP-SMZ, and extended-spectrum cephalosporins), and to retail poultry products (*12*). Moreover, similar results were obtained with retail poultry products and poultry carcasses from processing plants. This implies that drug-resistant poultry-source *E*. *coli* isolates originate in the birds, rather than being introduced from some exogenous reservoir later during the packaging and distribution process. This in turn suggests that on-farm practices, including use of antimicrobial agents for growth promotion, metaphylaxis, and therapy (*21*,*22*), may influence characteristics of *E*. *coli* that contaminate retail poultry products and, seemingly, are then transmitted to humans (*7*).

The greater overall similarity of drug-resistant human isolates to poultry isolates than to drug-susceptible human isolates applied not only to the hospital patient isolates compared with isolates from conventionally raised poultry, but also to the isolates from vegetarians compared with isolates from poultry raised with no antibiotics. This was surprising because the vegetarians ostensibly did not consume poultry and, therefore, should not have been directly exposed to poultry-source *E*. *coli*. However, this seeming paradox is consistent with the difficulty in confirming poultry consumption (along with most other individual-level exposures) as an epidemiologic risk factor for colonization with drug-resistant *E*. *coli* isolates among community-dwelling persons ([*23*]; J.R. Johnson, unpub. data). Assuming that the drug-resistant human isolates were derived from poultry, occurrence of poultry-source *E*. *coli* in both vegetarians and persons with conventional diets suggests that poultry-source drug-resistant *E*. *coli* may spread extensively through the human population without requiring individual exposure to poultry products. This suggestion would be consistent with evidence that household-level risk factors may be more predictive of colonization with drug-resistant *E*. *coli* than individual-level risk factors, and that household members often share *E*. *coli* clones with each another (*23*–*25*). The mechanisms for such diffusion, and methods to block the entry of such strains into the human population and their subsequent spread, need to be defined.

The virulence potential for humans of the present drug-resistant human and poultry *E*. *coli* isolates, which is related to their direct threat to human health, is unknown. Predictions regarding virulence potential await molecular comparisons with human clinical isolates (*9*,*10*,*12*) and experimental virulence assessment in vivo (*26*,*27*). Nonetheless, the abundance of ExPEC-associated virulence genes in some of these strains is of concern because it suggests a high likelihood of virulence. This would augment any health threat these strains may pose as passive vehicles for drug-resistance genes (*6*,*7*).

Potential limitations of this study warrant comment. Because we did not examine alternative sources for drug-resistant human isolates, we cannot exclude the possibility that other foods (*28*) or nonfood reservoirs (*29*) might yield even closer similarities to drug-resistant human isolates. Whether persons in the study consumed poultry products from the same lots or suppliers as those sampled is not known. Because the study was conducted in Minnesota and Wisconsin in mostly rural communities and with newly hospitalized patients and nonhospitalized vegetarians, generalizability of the results is unknown. We combined several resistance phenotypes because of low frequencies, which may have obscured differences. We also did not assess other molecular characteristics of strains, e.g., pulsed-field gel electrophoresis profiles (*12*), sequence types (*30*), and resistance elements (*28*). Use of multiple comparisons increased the likelihood of spurious associations (which we addressed by specifying a strict criterion for statistical significance), whereas the small sample size in certain subgroups reduced power for finding true associations.

Strengths of the study include substantial overall sample size, standardized concurrent processing of fecal and poultry samples, close matching of human and poultry samples, extensive molecular typing using virulence-relevant markers, and use of multiple analytical modalities. Additionally, we examined clinically relevant resistance phenotypes.

In summary, our findings suggest that in a contemporary US-based population, many human-source drug-resistant fecal *E*. *coli* isolates more likely originated in poultry than in humans, whereas drug-resistant poultry isolates likely derive from drug-susceptible poultry isolates. Our data extend this paradigm to clinically relevant agents other than fluoroquinolones, heighten concerns regarding the potential human health risk for antimicrobial drug use in poultry production, and suggest that avoidance of poultry consumption may not reliably provide personal protection.
